# Brain Tumor with Exhibit of Two Living Cases

**Published:** 1910-06

**Authors:** Wesley Taylor

**Affiliations:** Professor of Neurology and Therapeutics Atlanta School of Medicine, Atlanta, Ga.


### BRAIN TUMOR WITH EXHIBIT OF TWO LIVING CASES.

BY WESLEY TAYLOR, B. S., M. D.,

*Professor of Neurology and Therapeutics Atlanta School of Medicine, Atlanta, Ga.*

My first case is a carpenter aged 38, married and with seven healthy children. He has always been healthy and there is no suspicion or history of lues.

About June 1, 1908, he fell fifteen feet, striking on his back. Not until he got home did he notice that his head was slightly injured as well. For ten days he noticed some dizziness which was soon followed by headache in the left Rolandic region. This headache gradually increased and there was a tendency for him to fall in that direction. There was, however, no nausea and no symptoms on the part of his eyes or ears or any difficulty with swallowing or with speech.

On the 28th of June he went out in the morning to get a bucket of water. When he started back, he noticed that his right arm and leg were weak and that he could not use his arm for carrying. From this moment, the weakness increased rapidly and at the time of my visit on the 2nd of July he could not cross his right leg or raise his right arm to his face, though his left side was about normal. Muscular force was very much diminished in the arm and to a less extent also in his limb. Sensations likewise were lessened on the entire right side and all reflexes were increased. There was a sensitive spot over the Rolandic area found when one tapped with the finger on the left side of his head. His speech had begun to show hesitation and he soon became so tired he could not answer in an intelligent manner. I decided that it was necessary to bring him to a hospital, and that an operation was needed. On his arrival at the hospital, consultation was had with one of our leading surgeons who failed to see any necessity for an operation. As I insisted, another surgeon was called and they decided to put him on energetic mercural treatment. After he was put to bed and after treatment was begun, I was told that he improved and my diagnosis of a possible cyst or other rapidly growing tumor over the seat of tenderness on the left Rolandic region received a severe blow. I did not see him for some days, but at the end of the week called to see how he was progressing. As far as was possible for me to tell I concluded that he had become decidedly worse. So strong was this conviction that I called up the surgeons and requested another consultation. The first told me point blank that he thought an operation unnecessary. The second one said he was willing to operate the case, though he would do it on my advice and not because he believed it was necessary or that it would do any good. I told him that I would not accept this sort of charity and that if he had no confidence in the operation, he need not do it. To make a long matter short, I influenced the family to have the case turned over entirely and another surgeon was called. By this time he was unable to feed himself or to take any food except liquids. He had apparently ceased to understand what was said to him when he was addressed, and was indeed in a very miserable condition.

On August 11, he was operated on and a hemorrhagic cyst or encapsulated clot was found over the left Rolandic area, about the size of a kid glove orange. This had so flattened the convolutions of the brain that they could scarcely be seen. This cyst seemed to be enlarging and had increased the tension very much on the inside of the cranium. From now on recovery was remarkable. There were no complications of any kind and by the time he had recovered from the anaesthetic, he was able to talk and to move his arm and limb which, before the operation had been quite impossible. He sat up in a chair on the 15th, was walking around on the 19th and left the hospital on foot, on the 21st, and has been entirely well as far as is possible for me to see since that time. There is, I might add, some trouble with memory so he says, but this is a matter which is so slight that one might say recovery in this case was entirely perfect.

My next case is that of a school boy aged 12. As to family history, his father is living and well, mother died of tuberculosis at 25, one brother of pneumonia, while one sister age 14 is in good health. For past history he had measles at the age of five; is subject to cold, but has always been strong. At the age of six he fell down a flight of steps and struck his head. No permanent irregularity or bump was noticed on the skull at that time. About nine months ago, however, a lump was noticed on the left side of his head which gradually grew to its present size. About the middle of November what we might call the present illness began with nausea and vomiting, and it was noticed that he could not keep his fingers still, though he had good control of his lower limbs. He could walk, but when his foot was raised he could not measure distances. Then some slight movement was noticed about the arms. He began to lose flesh rapidly, and his eyes would roll from side to side. At the beginning as far as could be learned, he had no fever. Within ten days from the onset, he was taken to bed because he was no longer able to walk. Several days previously, however, it was noticed that his mind was becoming dull. By this time he would answer only when spoken to, and was slow with his replies. Soon after this, articulation became so poor that he could scarcely speak at all. His bowels were constipated from the start. When I first saw him in the hospital he was lying listlessly in bed, *perfectly relaxed* and emaciated in the extreme. He could not lift his head and could move his arms only with the greatest difficulty, and even then more from the shoulder than by virtue of the muscles of the arms proper. When he was spoken to he would only sometimes answer and that slowly and with an unintelligible grunt. He could swallow with the greatest difficulty but could not chew or masticate. Physical examination showed a lump on the left side of his head nearly as large as half a goose egg which seemed to cause him pain when it was handled. He had a temperature in the axilla of 104 2-10, and his pulse was running from 140 to 150. There was dullness of the right apex with rales. A marked tubercular reaction was easily obtained from the use of tuberculin ointment. A specimen of urine showed a very slight trace of albumin but without microscopic findings. Under these circumstances, it is not difficult to see what sort of a provisional diagnosis was made. This was pretty well concurred in by several physicians who examined him carefully. In cases of any cerebral growth it is my custom always to use some antiluetic treatment. As potassium iodide will usually increase trouble, where there is any suspicion of tuberculosis, I therefore in this case used inunctions of mercury. A surgeon was called at my request at the outset to see him but he was unable to come and examine the case for some days. By the time he arrived the boy had shown considerable improvement and an operation was not advisable. Recovery was so rapid and so pronounced that he was talking within five days. He entered the hospital on the 17th of December, and was referred to me about the 22nd. On the 3rd of January he was able to be propped up in bed and was sitting up out of bed on the 9th, and running around at will within three days more. He was discharged cured on the 29th, having almost regained his natural weight. His treatment consisted, besides the inunctions of mercury, in daily high colonic lavages. Later on he was put on an iron tonic. He was not given potassium iodide, but in its place succus alterans was used. Since that time he has been and remained entirely well and free from trouble of any kind and has regained his normal weight as well as accomplished the growth which is natural to a boy of his age.

While both of these cases are unusual in my opinion, they go to show the difficulty of diagnosis in brain troubles. In the first case there was very considerable difference of opinion as to the nature of the trouble, and naturally therefore regarding treatment. We had time enough fortunately, to enable us to thoroughly try out a strong anti-luetic treatment and this was done. In the second case there was, I believe, very little doubt in the minds of all of the physicians who really examined this boy, that we had to do with a tubercular process in the brain at least, and while time in this case was very precious, nevertheless, mercurials were employed and with results which have been outlined. This case was really as much, if not more difficult than the first one in its general aspects. A resume of these cases will show the exceeding difficulty of diagnosis, and should teach the necessity for a conservative diagnosis. At the same time, one dare not hesitate or delay operation in suitable cases too long. Should an operation be not necessary, if carefully done it will not accomplish any harm. Lastly, in all cases where cerebral growths are suspected one should immediately resort to energetic mercurial treatment.

Read before the Georgia State Medical Society, Athens, Ga., April 24th, 1910.

